# Circular RNA FOXP1 Induced by ZNF263 Upregulates U2AF2 Expression to Accelerate Renal Cell Carcinoma Tumorigenesis and Warburg Effect through Sponging miR-423-5p

**DOI:** 10.1155/2021/8050993

**Published:** 2021-09-03

**Authors:** Li Fang, Ting Ye, Yanmei An

**Affiliations:** ^1^Department of Nephrology, Wuhan Asia General Hospital, Wuhan City, Hubei Province, China; ^2^Department of Nephrology, Central Theater Command General Hospital of the Chinese People's Liberation Army, Wuhan City, Hubei Province, China; ^3^Department of Urology, Huai'an Second People's Hospital and the Affiliated Huai'an Hospital of Xuzhou Medical University, Huai'an, China

## Abstract

Renal cell carcinoma (RCC), as one of the most common malignant tumors in the urinary system, is featured with high morbidity and mortality. Although the improvement of clinical intervention, such as surgery technology, chemotherapy, and radiotherapy, has been made, the outcomes of RCC patients are still poor. Novel targets for RCC treatment are urgently needed. Recently, circRNA has been in-depth studied and is considered as a promising direction for gene target therapy. In this study, we explored the function of circFOXP1 in RCC progression and its underlying mechanisms. Firstly, we demonstrated the characterization and expression of circFOXP1 in RCC tissues and cells. Next, by conducting a serial experiment, we found that downregulated circFOXP1 inhibited cell proliferation, migration, invasion, and the Warburg effect. Next, our experiments found that circFOXP1 upregulated U2AF2 expression via sponging miR-423-5p in RCC cells. Moreover, we found that ZNF263 induced circFOXP1 expression in RCC cells. To sum up, our study partially demonstrated that the novel ZNF263/circFOXP1/miR-423-5p/U2AF2 axis has a role in RCC progression. Our results might provide a new direction for RCC therapeutic target exploring.

## 1. Introduction

Renal cell carcinoma (RCC) is considered one of the most common malignant tumors in urinary cancers. It has been reported that more than 40,000 patients were firstly diagnosed with RCC in 2018 worldwide [1]. Although considerable improvements in diagnosis strategy and clinical therapy have been made in the past decades, the morbidity and mortality of RCC are still rising[2]. Most patients were diagnosed at a metastasis stage due to fact that the initial symptoms of RCC are hardly being noticed [3]. Furthermore, RCC is considered chemotherapy- or radiotherapy-resistant tumor, which brought enormous clinical intervention. Finding novel therapeutic targets for RCC is urgently needed.

Circular RNAs (circRNAs) are defined as one subtype of noncoding RNAs characterized by their circular configuration structure by connecting its 5′ and 3′ ends [4]. With the high-throughput technology innovation in the past decades, the function of circRNAs has been investigated in-depth. Mechanically, circRNAs are capable of playing their functions via transcriptionally regulating gene expression, interacting with proteins, and encoding protein [5–7]. Biologically, circRNAs were reported to participate in various cellular progressions, including immune response, viral infection, inflammation, angiogenesis, and tumorigenesis [8–11]. The function of circRNAs in RCC development has been widely investigated. Xue et al. revealed that circ-AKT3 was involved in the progression of RCC through modulating miR-296-3p/E-cadherin signals [12]. Li et al. demonstrated that circTLK1 promotes RCC progression via sponging miR-136-5p [13]. Li et al. investigated the role of circPRRC2A in RCC progression [14]. Emerging evidences suggest the essential role of circRNAs in RCC development, which indicates that circRNA is one promising therapeutic target for RCC. Our group noticed that circRNA FOXP1 was involved in multiple tumorigenesis, such as gallbladder cancer, hepatocellular carcinoma, and lung cancer [15–17], while whether circFOXP1 exerts its role in RCC progression remains uncovered.

In the current study, we hypothesized that circFOXP1 plays its role in the progression of RCC. By elucidating the characterization and expression of circFOXP1 in RCC tissues and cells, our results found that circFOXP1 expression was upregulated in RCC tumors. By conducting a serial biological experiment, we found that downregulated circFOXP1 inhibited cell proliferation, migration, invasion, and the Warburg effect. Furthermore, we utilized bioinformatics analysis, RNA pull-down assay, RIP, and luciferase reporter gene assay, and we found a novel circFOXP1/miR-423-5p/U2AF2 axis. Moreover, the upstream regulator of circFOXP1 was verified; ZNF263 induced circFOXP1 expression in RCC cells. Collectively, our results suggested that circFOXP1 induced by ZNF263 promotes RCC progression through the miR-423-5p/U2AF2 axis. We might find novel diagnostic or therapeutic targets for RCC.

## 2. Methods and Materials

### 2.1. Tissue Samples

Renal cell carcinoma tissue samples (thirty cases) and their adjacent normal tissues came from patients who experienced operation at Huai'an Second People's Hospital and the Affiliated Huai'an Hospital of Xuzhou Medical University from 2018 to 2019. Tissues were frozen at -80°C immediately in liquid nitrogen for further investigation. Before surgery, patients had not received any chemotherapy, radiotherapy, or gene target therapy. Informed consent was obtained from patients before this study. All tissues were confirmed by three pathologists independently. The Ethics Committee of Huai'an Second People's Hospital and the Affiliated Huai'an Hospital of Xuzhou Medical University approved all approaches in this study.

### 2.2. Cell Culture and Transfection

All cells applied in this research (HK-2, 293 T, ACHN, 786-O, OSRC-2, A 498, and CAKI-1) were commercially acquired from American Type Culture Collection (ATCC, USA). Cells were cultured in a humid environment with 5% CO_2_ at 37°C. Medium containing Dulbecco's modified Eagle's medium (DMEM, USA) and 10% fetal bovine serum (FBS, USA) was used to culture cells. All shRNAs, lentiviruses, plasmids, and probes used in the current study were synthesized and procured from GeneChem company (Shanghai). Transfection was performed using Lipofectamine 3000 kit (Invitrogen, USA) according to the manufacturer's protocol.

### 2.3. RNA Isolation and Real-Time PCR

According to the producer's protocol, total RNAs were harvested using TRIzol reagent (Invitrogen) and bred with RNAse R (Epicentre Technologies, USA) at 37°C for half an hour. PrimeScript RT reagent Kit (Takara, Shiga, Japan) was used to accomplish reverse transcription of RNAs following the manufacturer's protocol. Quantitative RT-PCR was conducted applying a real-time PCR system (Applied Biosystems) and T.B. Green Premix Ex Taq II (Takara, Japan). GAPDH or U6 normalized relative expression of RNAs by using the 2-*ΔΔ*CTmethod. Primers used in this study are as follows (Table 1).

### 2.4. Cell Proliferation Detection

The proliferation rate of cells was assessed by the CCK-8 reagent purchased from Dojindo (Kumamoto, Japan). Cells were inoculated into each well of a 96-well plate, and 10 liters of CCK-8 reagent was attached to each well at the set time (24 hours, 48 hours, 72 hours, and 96 hours). After 2 h incubation, absorbance was estimated using a microplate reader (BioTek, USA) at 450 nm.

### 2.5. Cell Migration and Invasion Detection

The migration and invasion ability of cells was determined by transwell assay. We put the crosshole analytical insert (Millipore, USA) in a 24-well plate. All medium and reagents were incubated at 37°C. In the above transwell chamber, the normal layer was used to perform the migration assay, while the Matrigel-coated membrane (BD Biosciences) was applied to test the invasion ability. First, we put 600 μl of serum-free RPMI 1640 (containing 10% fetal bovine serum) into the bottom container. After that, we inoculated 10,000 cells into the upper chamber of 200 L RPMI 1640. After culturing in an incubator for 48 hours, cells were fixed within the membrane with methanol and applied with crystal violet for 15 minutes. Subsequently, cells were visualized under a microscope, and cell counts were calculated in five different areas.

### 2.6. Warburg Effect Level Detection

For lactate assessment, the lactate concentration in cell lysis was assessed using a lactate assay kit (BioVision, USA) following the manufacturer's instructions. For glucose uptake testing, the treated cells were cultured with 100 M NBDG (11,046, Cayman) for 30 minutes before washing with ice-cold PBS. After that, we accounted for the fluorescence of FL-1 in accordance with the manufacturer's instructions. As for ATP detection, an ATP detection kit (ab113849, ABCAM) was applied to measure ATP inside the treated cells by measuring the luciferase activity.

### 2.7. Western Blotting

According to the manufacturer's scheme, a protein extraction kit (Key Gene, China) was used to isolate protein from stably transfected cells. The protein qualification was detected by a BCA kit (Pierce, USA). Proteins were isolated by electrophoresis exerting polyacrylamide gels with SDS and separated by polyvinylidene fluoride (PVDF) membrane. Then, 5% skimmed milk powder was used to block the membrane in TBST buffer for 90 min, and the membrane was incubated with the primary antibody at 4°C overnight. Then, the membrane was washed using TBST buffer solution three times and then subjected to secondary antibodies for 2 hours at room temperature. Results were visualized using an enhanced chemiluminescence system (Millipore, USA). The Image Lab Software was used to analyze the related data. The antibodies used in this study are as follows: U2AF2 (Proteintech; 1 : 2000; #15624) and actin (Abcam; 1 *μ*g/ml; ab8226).

### 2.8. RNA Pull-Down

A total of 10^7^ RCC cells were collected, dissolved, and ultrasonically treated. Probe-coated beads were formatted by subjecting C-1 magnetic beads (Life Technologies) to probes at 25 degrees for 100 min. Cell lysate with the indicated probe was cultured at 4°C for 12 hours. After washing with washing buffer, the RNA mixture bound to the beads was isolated by RT-qPCR with RNA mini kit (QIAGEN).

### 2.9. AGO2-RIP

RIP assay was conducted using an RNA-binding protein immunoprecipitation kit (Millipore) according to the manufacturer's instructions. Results were analyzed using the qRT-PCR assay. The experiment was conducted in triplicate.

### 2.10. Luciferase Reporter Gene Assay

The wild type (wt) or mutant type (Mut) of RNA sequences were constructed by GeneChem company (Shanghai) and then inserted into indicated luciferase reporter gene plasmids. We transfected the reporter plasmid into RCC cells using lipofectamine 3000. Subsequently, mimics and treated luciferase reporter gene plasmids were coinfected into cells as indicated. Results were detected using a Dual-Luciferase Reporter System Kit (Promega, USA) following the manufacturer's protocol. The experiment was performed at least three times.

### 2.11. Statistical Analysis

We conducted these experiments in triplicate, and the data were expressed using the standard deviation of the mean value. Data analysis was utilized using an SPSS 22.0 (SPSS, USA) system. The difference between groups was analyzed by Students' *T*-test. The difference among three or multiple groups was calculated using one-way ANOVA test. *P* < 0.05 was considered statistically significant.

## 3. Results

### 3.1. Expression and Characterization of circFOXP1 in RCC

**Figure 1:**
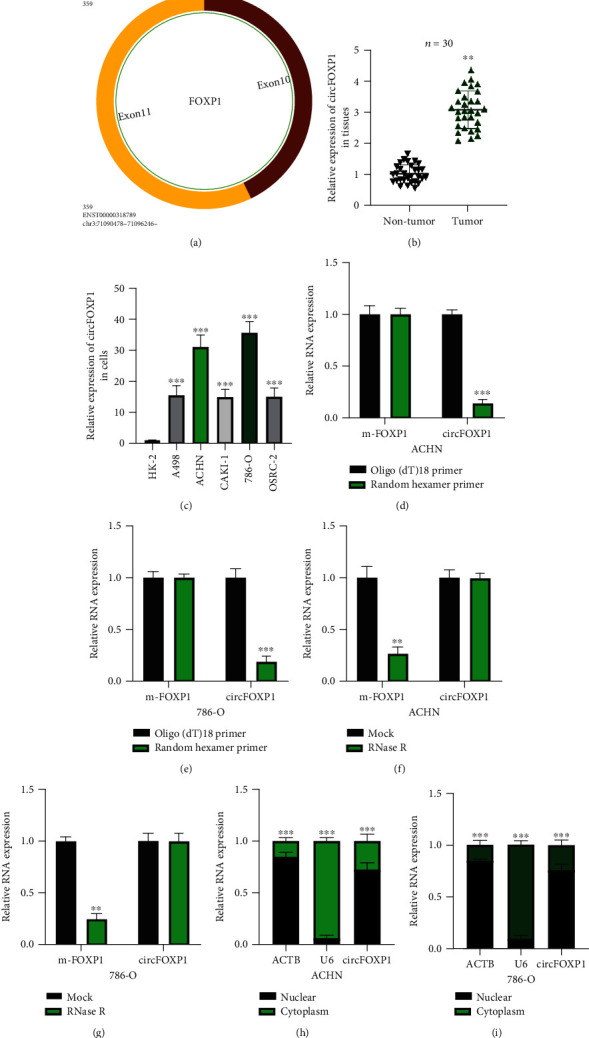
Characterization of circFOXP1 in RCC. (a) The exonic information about circFOXP1 was elucidated using the circBase dataset (http://www.circbase.org/). (b) Expression level of circFOXP1 in thirty pairs of RCC tissue samples was measured. (c) Expression level of circFOXP1 in RCC cell lines and normal renal cell HK-2 was tested. (d, e) Random hexamer primers were applied, and the results were analyzed using qRT-PCR in ACHN (d) and 786-O cells (e). (f, g) ACHN (f) and 786-O (g) cells were under actinomycin D treatment; relative circFOXP1 and liner FOXP1 (m-FOXP1) expression was detected using qRT-PCR. (h, i) Expression levels of circFOXP1 in the cytoplasm or nucleus of ACHN (h) or 786-O (i) cells were detected by qRT-PCR after cellular RNA fractionation. All experiments were repeated in triplicate, ^∗∗^*P* < 0.01 and ^∗∗∗^*P* < 0.001.

To explore whether circFOXP1 was involved in the progression of RCC, firstly, we verified the characterization and expression of circFOXP1 in RCC tissues and cells. The exonic information about circFOXP1 was acquired (Figure 1(a)). Next, we found that circFOXP1 expression was significantly upregulated in tumor tissues compared with adjacent normal tissues (Figure 1(b)). The upregulation tendency was also found in RCC cell lines (Figure 1(c)). Previous studies indicated that dysregulation of circFOXP1 might be involved in the progression of tumorigenesis. Therefore, we presumed that circFOXP1 plays its role in RCC development. Circular RNA characterizations of circFOXP1 in RCC cells were tested. As shown in Figures 1(d) and 1(e), circFOXP1 expression was obviously decreased in cells upon random hexamer primer treatment, but not oligo (dT)18 primer, suggesting that circFOXP1 has no poly-A-tail. Moreover, we found that circFOXP1 was resistant to RNase R digestion, but not its liner form mFOXP1 (Figures 1(f) and 1(g)). Subsequently, we found that circFOXP1 was mainly located in the RCC cell cytoplasm.

### 3.2. Downregulated circFOXP1 Inhibited RCC Proliferation, Migration, and Invasion and Decreased Warburg Effect

**Figure 2:**
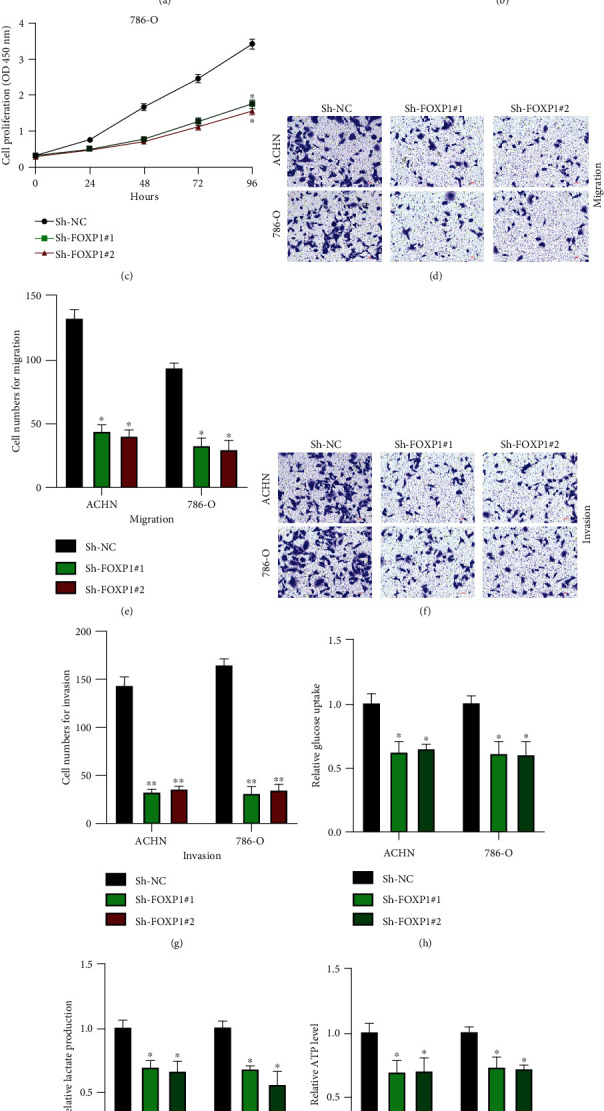
Downregulated circFOXP1 inhibited RCC proliferation, migration, and invasion and decreased Warburg effect. (a) Cells were treated with Sh-NC, Sh-FOXP1#1, or Sh-FOXP1#2 as indicated; the expression level of circFOXP1 and mFOXP1 was measured by qRT-PCR. (b, c) Cell proliferation levels were measured using CCK-8 assay in ACHN (b) and 786-O (c) cells. (d, e) Cell migration levels were detected using transwell migration assay (d); a comparative analysis was shown (e). (f, g) Cell invasion levels were assessed by transwell invasion assay (f); a comparative analysis was calculated (g). (h–j) Expression change of glucose (h), lactate(i), and ATP (j) in ACHN and 786-O cells was detected. Experiments were repeated three times, ^∗^*P* < 0.05 and ^∗∗^*P* < 0.01.

Here, we explored the biological functions of circFOXP1 in RCC cellular progression. Firstly, we generated circFOXP1 knockdown cell models as indicated (Figure 2(a)). Expression level of circFOXP1 in ACHN and 786-O cells was efficiently downregulated; the liner form of FOXP1 remained the same. As shown in Figures 2(b) and 2(c), downregulated circFOXP1 suppressed ACHN and 786-O cell proliferation levels. Additionally, downregulated circFOXP1 inhibited cell migration (Figures 2(d) and 2(e)) and invasion (Figures 2(f) and 2(g)) levels. Furthermore, we found that the Warburg effect in circFOXP1 knockdown ACHN and 786-O cells was inhibited (Figures 2(h)–2(j)).

### 3.3. circFOXP1 Sponges miR-423-5p

**Figure 3:**
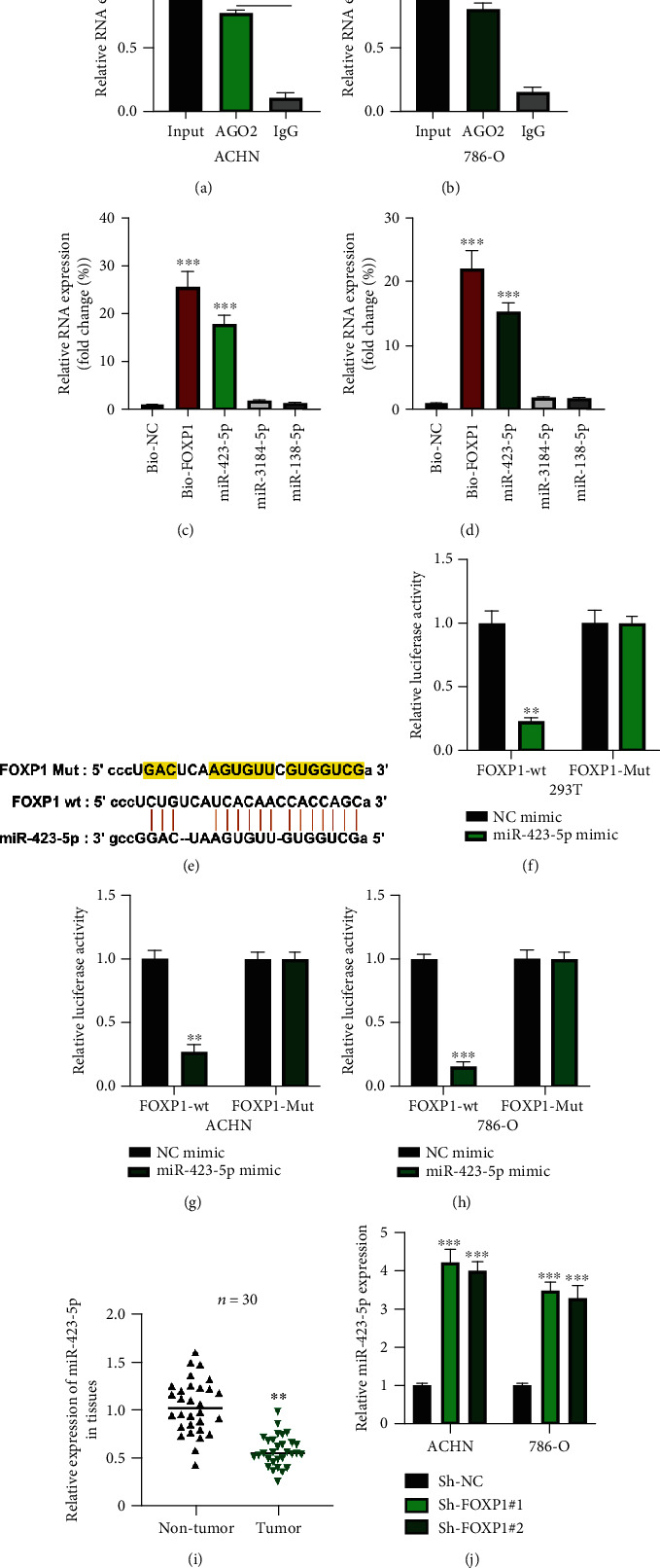
circFOXP1 sponges to miR-423-5p. (a, b) AGO2-RIP was utilized in ACHN (a) and 786-O (b) cells; relative circFOXP1 was measured by qRT-PCR. (c, d) Bioinformatics prediction of circFOXP1 targets was performed using the ENCORI dataset (http://starbase.sysu.edu.cn/) with CLIP Data: medium stringency (≥2), and Degradome Data: low stringency ≥1). Results were tested using biotinylated RNA pull-down; relative RNA expression was measured by qRT-PCR in ACHN (c) and 786-O cells (d). (e) Predicted circFOXP1 wild type (wt) or mutant type (Mut) bind sequences with miR-423-5p. (f–h) Relative luciferase activities in reporter vector-treated 293T (f), ACHN (g), and 786-O (h) cells were measured as indicated. (i) Expression level of miR-423-5p in thirty pairs of RCC tissues was determined by qRT-PCR assay. (j) Expression level of miR-423-5p in circFOXP1 downregulated ACHN, and 786-O cells were measured by qRT-PCR. All assays were conducted at least three times, ^∗∗^*P* < 0.01 and ^∗∗∗^*P* < 0.001.

The molecular mechanisms underlying the function of circFOXP1 in RCC progression were investigated. RIP assay with anti-AGO2 and anti-IgG antibodies was conducted to assess the miRNA binding ability of circFOXP1. As shown in Figures 3(a) and 3(b), circFOXP1 was abundantly enriched in anti-AGO2-bound antibodies. Next, the bioinformatics analysis result showed us three potential downstream targets for circFOXP1. Biotinylated RNA pull-down experiments showed us that miR-423-5p was abundantly enriched in bio-circFOXP1 probes (Figures 3(c) and 3(d)). Predicted binding sequences were presented (Figure 3(e)). The interaction between circFOXP1 and miR-423-5p was confirmed by utilizing luciferase reporter gene assay (Figures 3(f)–3(h)). Next, expression levels of miR-423-5p in thirty pairs of RCC tissues were determined; we found that miR-423-5p was decreased in tumor tissues compared with normal healthy tissues (Figure 3(i)). Furthermore, the expression level of miR-423-5p in ACHN and 786-O cells was negatively regulated by circFOXP1 (Figure 3(j)).

### 3.4. U2AF2 Is a Downstream Target of miR-423-5p

**Figure 4:**
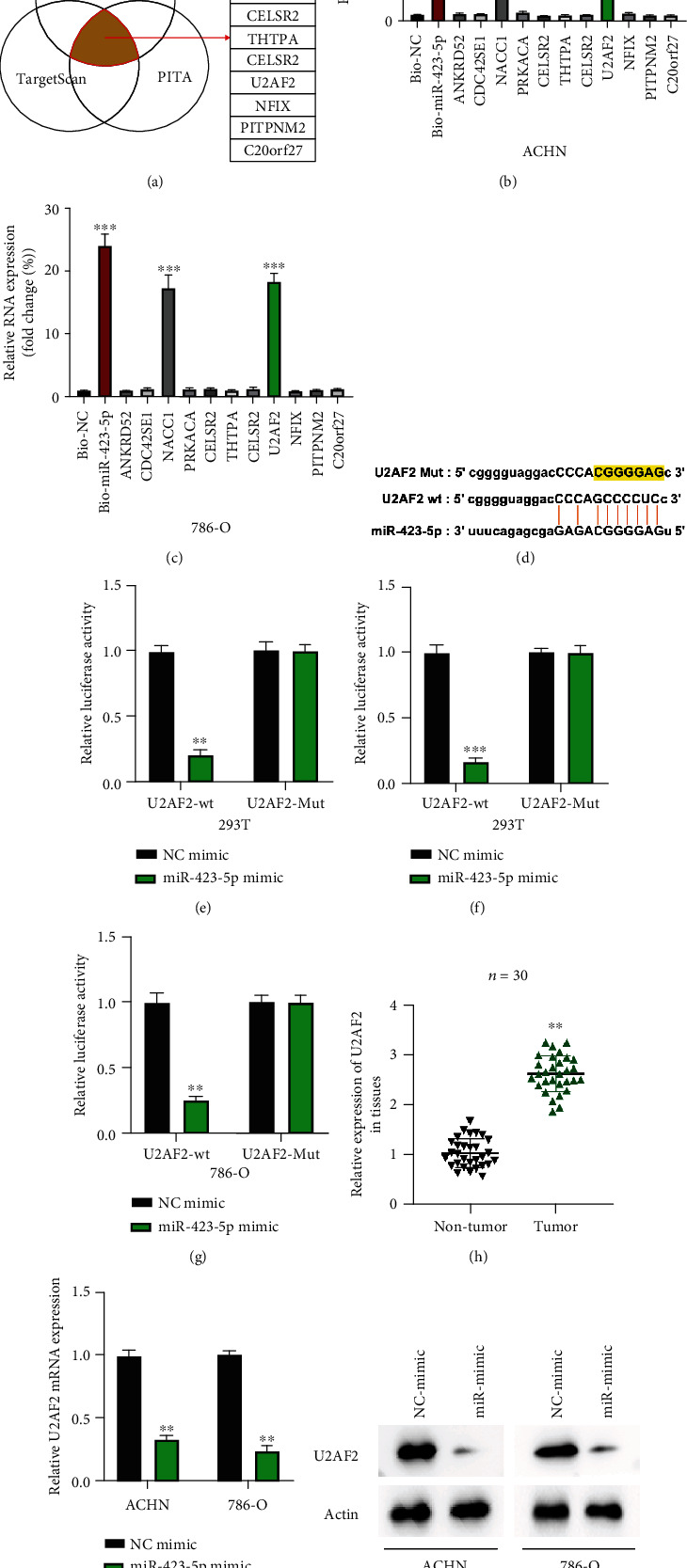
U2AF2 is a downstream target of miR-423-5p. (a) Downstream targets for miR-423-5p were predicted using the DIANA tool microT (http://diana.imis.athena-innovation.gr/DianaTools/index.php?r=microT_CDS/index), PITA (https://genie.weizmann.ac.il/pubs/mir07/mir07_data.html), and TargetScan (http://www.targetscan.org/mamm_31/) datasets with CLIP Data: strict stringency (≥5), and Degradome Data: medium stringency (≥2); results were presented by Venn diagram. (b, c) Putative targets of miR-423-5p were examined by biotinylated RNA pull-down in ACHN (b) and 786-O (c) cells; results were measured using the qRT-PCR assay. (d) Predicted U2AF2 wild type (wt) or mutant type (Mut) bind sequences with miR-423-5p. (e–g) Relative luciferase activities in indicated reporter vector-treated 293T (e), ACHN (f), and 786-O (g) cells were measured. (h) Expression levels of U2AF2 in thirty pairs of RCC tissues were measured by qRT-PCR assay. (i, j) Expression levels of U2AF2 mRNA (h) or protein (i) in miR-423-5p overexpression ACHN and 786-O cells were measured by qRT-PCR and western blot. All assays were conducted in triplicate, ^∗∗^*P* < 0.01 and ^∗∗∗^*P* < 0.001.

Subsequently, bioinformatics prediction found us eleven putative targets for miR-423-5p. RNA pull-down using bio-miR-423-5p and bio-NC probes showed us that NACC1 and U2AF2 were abundantly enriched in bio-miR-423-5p probes compared with others in ACHN and 786-O cells (Figures 4(b) and 4(c)). The interaction between miR-423-5p and NACC1 was per published researches [18, 19]. Here, we aimed to investigate the relation between miR-423-5p and U2AF2. Predicted binding sequences between miR-423-5p and U2AF2 are presented in Figure 4(d). Luciferase reporter gene assay performed in 239T, ACHN, and 786-O cells confirmed that miR-423-5p directly targets U2AF2 (Figures 4(e)–4(g)). Subsequently, we found that U2AF2 expression in RCC tumor tissues was increased compared with normal healthy tissues (Figure 4(h)). Moreover, we found that miR-423-5p overexpression inhibited U2AF2 expression in RCC cells (Figures 4(i) and 4(j)).

### 3.5. circFOXP1 Regulates U2AF2 Expression to Promote RCC Progression through Sponging miR-423-5p

**Figure 5:**
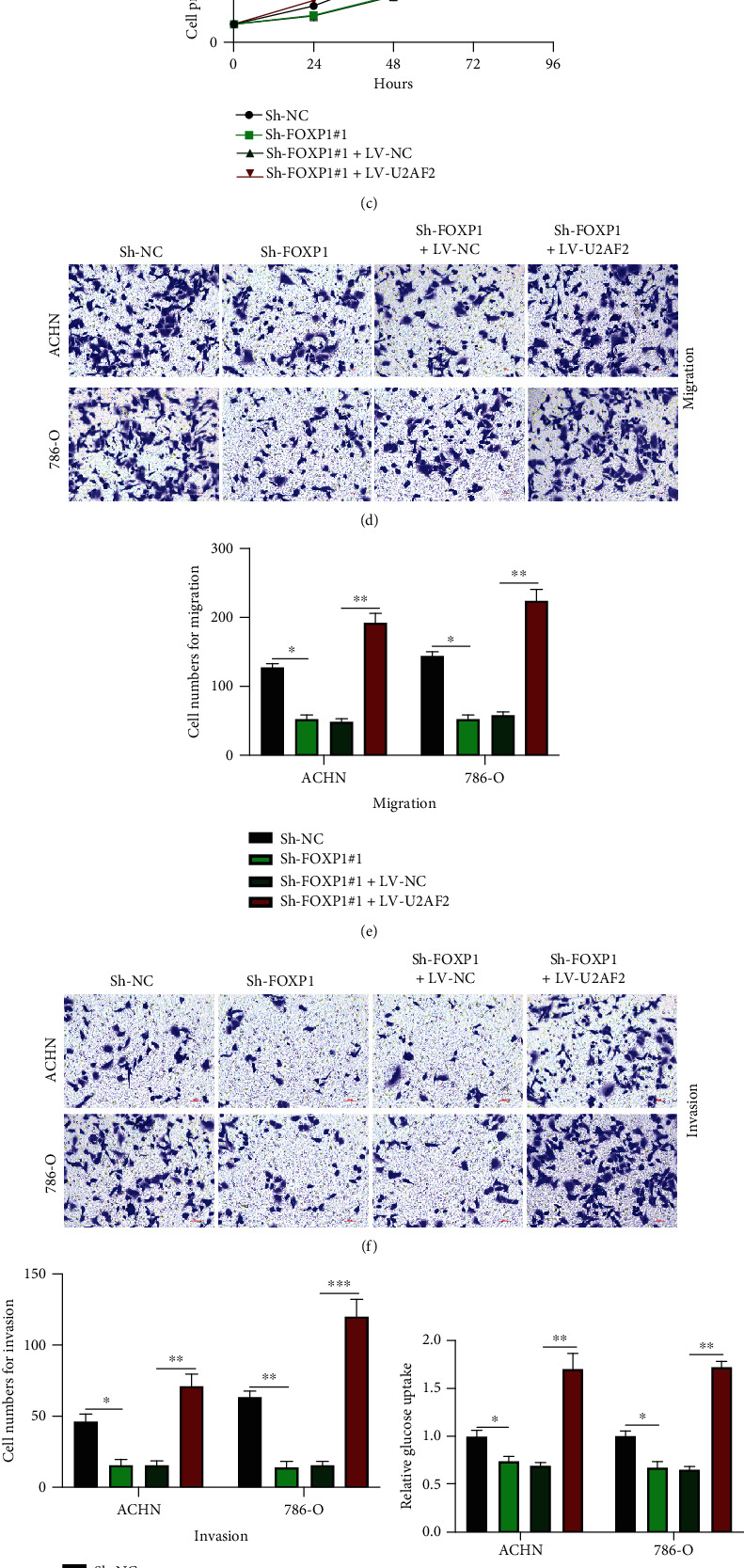
circFOXP1 regulates U2AF2 expression to promote RCC progression through sponging miR-423-5p. (a) ACHN and 786-O cells were upon different treatment as indicated; the expression level of U2AF2 was measured. (b, c) CCK-8 assay was applied to measure cell proliferation levels of treated as indicated ACHN (b) and 786-O (c) cells. (d, e) Transwell migration assay was performed to evaluate cell migration abilities (d); results were statistically analyzed (e). (f, g) Transwell invasion assay was conducted to measure cell invasion levels (f); comparative analysis of results was shown (g). (h–j) Expression change of glucose (h), lactate (i), and ATP (j) in ACHN and 786-O cells was measured. All assays were conducted in triplicate, ^∗^*P* < 0.05, ^∗∗^*P* < 0.01, and ^∗∗∗^*P* < 0.001.

According to the above results, we found that circFOXP1 modulated RCC cellular progression and Warburg effect and a novel circFOXP1/miR-423-5p/U2AF2 axis. To determine whether circFOXP1 plays its role through the miR-423-5p/U2AF2 axis, as shown in Figure 5(a), it was indicated that cell models were constructed. By conducting CCK-8 (Figures 5(b) and 5(c)), transwell migration (Figures 5(d) and 5(e)), and transwell invasion assays (Figures 5(f) and 5(g)), we found that the inhibitive effects of circFOXP1 knockdown on RCC cellular behavior were reversed by U2AF2 overexpression. Furthermore, downregulated circFOXP1 inhibited the RCC cell Warburg effect but was rescued by U2AF2 overexpression (Figures 5(h)–5(j)). Our results suggested that circFOXP1 promoted U2AF2 expression to accelerate RCC progression through sponging miR-423-5p.

### 3.6. Expression of circFOXP1 Is Induced by ZNF263

**Figure 6:**
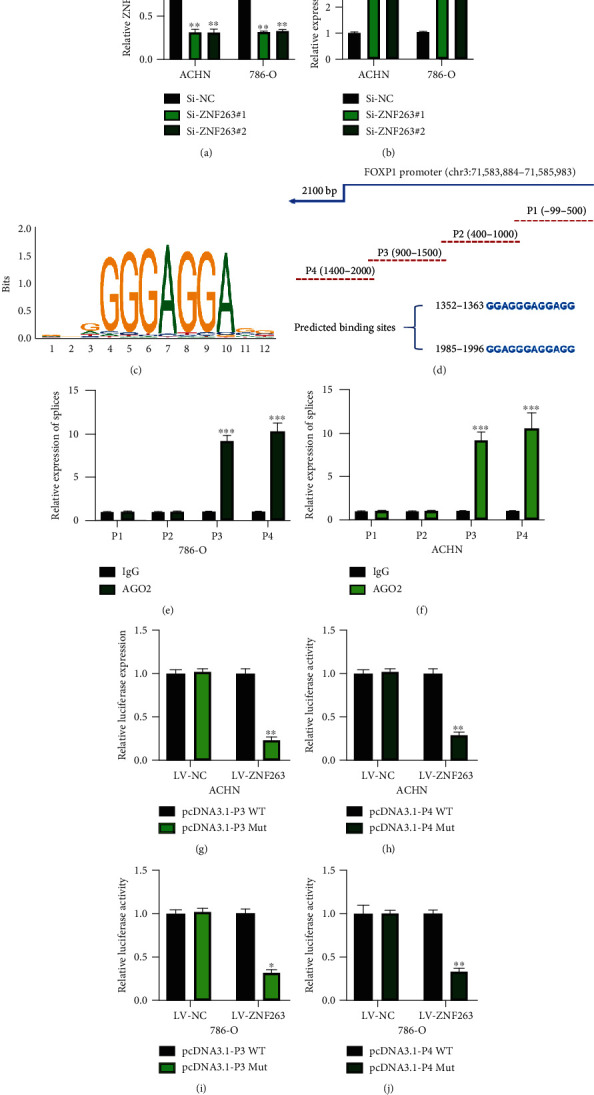
Expression of circFOXP1 is induced by ZNF263. ACHN and 786-O cells were infected with Si-NC, Si-ZNF263#1, and Si-ZNF263#2. (a) Expression of ZNF263 in treated as indicated cells was measured using qRT-PCR. (b) Expression of circFOXP1 in ZNF263 knockdown cells was measured by qRT-PCR. (c) Bioinformatically predicted binding sequence of ZNF263. (d) Bioinformatically predicted binding site of FOXP1 promoter splices. (e, f) AGO2-RIP was conducted to verify the binding ability between ZNF263 and FOXP1 promoter splices in ACHN (e) and 786-O (f). (g–j) Luciferase reporter assay was conducted to confirm the interaction between FOXP1 promoter splices (P3 and P4) and ZNF263 in ACHN (g and i) and 786-O cells (h and j). All experiments were performed in triplicate, ^∗^*P* < 0.05, ^∗∗^*P* < 0.01, and ^∗∗∗^*P* < 0.001.

Utilizing the bioinformatics method (NCBI: https://www.ncbi.nlm.nih.gov/, UCSC: http://genome.ucsc.edu/, and JASPAR: http://jaspar.genereg.net/), we found that ZNF263 is one promising upstream regulator of FOXP1 mRNA. After we generated ZNF263 knockdown cell models (Figure 6(a)), we found that FOXP1 circRNA was negatively regulated by ZNF263 in RCC cells (Figure 6(b)). Bioinformatics prediction results suggested that the FOXP1 promoter has two putative binding sites to ZNF263 (Figures 6(c) and 6(d)). The prediction results were verified using the AGO2-RIP assay (Figures 6(e) and 6(f)). Furthermore, we conducted a luciferase reporter assay to confirm the interaction between FOXP1 promoter and ZNF263 in RCC cells. It was found that ZNF263 was able to interact with two splices (P3 and P4 as indicated) of the FOXP1 mRNA promoter. Our results suggested that ZNF263 could regulate circFOXP1 expression in RCC cells.

## 4. Discussion

The incidence of RCC accounts for about 3% of malignancies worldwide [20]. Unfortunately, even if patients received surgical resection and radiotherapy, 30% of patients develop into metastatic stage [21, 22]. In recent years, emerging evidence suggests that multiple gene mutations contribute to the initiation and progression of RCC, while the underlying molecular mechanisms of RCC progression are not fully understood.

In this study, we focused on the role of circFOXP1 in RCC progression. The characterization and expression of circFOXP1 were verified. circFOXP1 was found to be upregulated in RCC tissues and cell lines. Based on that, we generated circFOXP1 knockdown cell models and investigated their cellular functions. By conducting CCK-8 and transwell assays, we found that downregulated circFOXP1 inhibited RCC cell proliferation, migration, and invasion. Our results suggest that circFOXP1 acts as one oncogene in RCC progression.

As we knew, during tumorigenesis, cancer cells have to rewire its metabolism to keep themselves alive and reproducing. The phenomenon that cancer cells change their glucose uptake and fermenting into lactate is called the Warburg effect [23–25]. In the past decade, the role of the Warburg effect has been investigated in-depth. The Warburg effect contributes to the cellular progression of cancer cells [26], relates to the initiation and progression of inflammation diseases [27], participates in immunologic response [28], and is involved in drug resistance [29]. The essential roles of the Warburg effect in various cellular progressions have been verified. One published research has demonstrated that circFOXP1 exerts its function in tumorigenesis via regulating the Warburg effect [16]. Here, our results found that downregulated circFOXP1 inhibited the Warburg effect in RCC cells, which might partially elucidate the impact of circFOXP1 on RCC cell proliferation, migration, and invasion.

Next, by utilizing bioinformatics analysis and a serial experiment, we found that miR-423-5p is one downstream target for circFOXP1, and circFOXP1 promoted U2AF2 expression in RCC cells via sponging miR-423-5p. Li et al. have elucidated that U2AF2 contributes to non-small-cell lung cancer partially via modulating the Warburg effect [30]. Our study found that circFOXP1 modulates RCC cell proliferation, migration, invasion, and Warburg effect through the miR-423-5p/U2AF2 axis. Furthermore, ZNF263 was found to be an upstream regulator of circFOXP1 in RCC cells.

Although the role of ZNF263/circFOXP1/miR-423-5p/U2AF2 axis has been partially elucidated in RCC progression, our study still demands further investigation. For example, we need more tissue samples to further to confirm the expression tendency of circFOXP1 in RCC. Moreover, the downstream pathway of U2AF2 in RCC cellular progression and the Warburg effect phenomenon need to be elucidated.

Collectively, our study partially demonstrated the role of circFOXP1 in RCC progression and demonstrated its underlying molecular mechanisms. Our results suggest that circFOXP1 is one promising diagnosis and therapeutic target for RCC.

## Figures and Tables

**Table 1:** 

Gene		Sequence
circFOXP1	Forward	5′-CTCCTCTGCACCTTCCAAGA-3′
	Reverse	5′-ATCATAGCCACTGACACGGG-3′
mFOXP1	Forward	5′-CTTGCTCAAGGCATGATTCC-3′
	Reverse	5′-CCTTGGTTCGTCAGCCAGTA-3′
miR-423-5p	Forward	5′-CGAAGTTCCCTTTGTCATCCT-3′
	Reverse	5′-GTGCAGGGTCCGAGGTATTC-3′
miR-3184-5p	Forward	5′-TGAGAAACCTCAGATTGAGCTTTT-3′
	Reverse	5 ′-CTCTACAGCTATATTGCCAGCCA-3′
miR-138-5p	Forward	5′-GCGAGCTGGTGTTGTGAATC-3′
	Reverse	5′-AGTGCAGGGTCCGAGGTATT-3′
U2AF2	Forward	5′-TATGTGCCTGGGGTTGTGTC-3′
	Reverse	5′-TGGCATTCTTGGCTCCCAC-3′
GAPDH	Forward	5′-TTCCGTGTCCCCACTGCCAACGT-3′
	Reverse	5′-CAAAGGTGGAGGAGTGGGTGTCGC-3′

## Data Availability

The data performed to support the findings of this study are included within the article.

## Abbreviations

CircRNAs: Circular RNAs
FOXP1: Forkhead Box P1
U2AF2: U2 small nuclear RNA auxiliary factor 2
RCC: Renal cell carcinoma
ZNF263: Zinc finger protein 263
CCK-8: Cell Counting Kit 8
mRNA: Massage RNA
RIP: RNA-binding protein immunoprecipitation
AGO2: Argonaute RISC catalytic component 2.

## References

[1] Bray F., Ferlay J., Soerjomataram I., Siegel R. L., Torre L. A., Jemal A. (2018). Global cancer statistics 2018: GLOBOCAN estimates of incidence and mortality worldwide for 36 cancers in 185 countries. *CA: A Cancer Journal for Clinicians*.

[2] Capitanio U., Montorsi F. (2016). Renal cancer. *The Lancet*.

[3] Cohen H. T., McGovern F. J. (2005). Renal-cell carcinoma. *New England Journal of Medicine*.

[4] Hentze M. W., Preiss T. (2013). Circular RNAs: splicing's enigma variations. *The EMBO Journal*.

[5] Chen L. L. (2016). The biogenesis and emerging roles of circular RNAs. *Nature reviews. Molecular cell Biology*.

[6] Dong Y., He D., Peng Z. (2017). Circular RNAs in cancer: an emerging key player. *Journal of Hematology & Oncology*.

[7] Zhang Y., Xue W., Li X. (2016). The biogenesis of nascent circular RNAs. *Cell Reports*.

[8] Lei M., Zheng G., Ning Q., Zheng J., Dong D. (2020). Translation and functional roles of circular RNAs in human cancer. *Molecular Cancer*.

[9] Liu Y., Yang Y., Wang Z. (2020). Insights into the regulatory role of circrna in angiogenesis and clinical implications. *Atherosclerosis*.

[10] Marques-Rocha J. L., Samblas M., Milagro F. I., Bressan J., Martinez J. A., Marti A. (2015). Noncoding RNAs, cytokines, and inflammation-related diseases. *The FASEB Journal*.

[11] Yan L., Chen Y. G. (2020). Circular RNAs in immune response and viral infection. *Trends in Biochemical Sciences*.

[12] Xue D., Wang H., Chen Y. (2019). Circ-AKT3 inhibits clear cell renal cell carcinoma metastasis via altering miR-296-3p/E-cadherin signals. *Molecular Cancer*.

[13] Li J., Huang C., Zou Y., Ye J., Yu J., Gui Y. (2020). Circtlk1 promotes the proliferation and metastasis of renal cell carcinoma by sponging miR-136-5p. *Molecular Cancer*.

[14] Li W., Yang F. Q., Sun C. M. (2020). circPRRC2a promotes angiogenesis and metastasis through epithelial-mesenchymal transition and upregulates TRPM3 in renal cell carcinoma. *Theranostics*.

[15] Li O., Kang J., Zhang J. J. (2020). Circle RNA FOXP1 promotes cell proliferation in lung cancer by regulating miR-185-5p/Wnt1 signaling pathway. *European Review for Medical and Pharmacological Sciences*.

[16] Wang S., Zhang Y., Cai Q. (2019). Circular RNA FOXP1 promotes tumor progression and Warburg effect in gallbladder cancer by regulating PKLR expression. *Molecular Cancer*.

[17] Wang W., Li Y., Li X. (2020). Circular RNA circ-FOXP1 induced by SOX9 promotes hepatocellular carcinoma progression via sponging miR-875-3p and miR-421. *Biomedicine & Pharmacotherapy*.

[18] Du W., Feng Z., Sun Q. (2018). LncRNA linc00319 accelerates ovarian cancer progression through miR-423-5p/NACC1 pathway. *Biochemical and Biophysical Research Communications*.

[19] Tao H. F., Shen J. X., Hou Z. W., Chen S. Y., Su Y. Z., Fang J. L. (2020). LncRNA FOXP4‑AS1 predicts poor prognosis and accelerates the progression of mantle cell lymphoma through the miR‑423‑5p/NACC1 pathway. *Oncology Reports*.

[20] Scelo G., Larose T. L. (2018). Epidemiology and risk factors for kidney cancer. *Journal of Clinical Oncology*.

[21] Blanco A. I., Teh B. S., Amato R. J. (2011). Role of radiation therapy in the management of renal cell cancer. *Cancers*.

[22] De Meerleer G., Khoo V., Escudier B. (2014). Radiotherapy for renal-cell carcinoma. *The Lancet Oncology*.

[23] Potter M., Newport E., Morten K. J. (2016). The Warburg effect: 80 years on. *Biochemical Society Transactions*.

[24] Vander Heiden M. G., Cantley L. C., Thompson C. B. (2009). Understanding the Warburg effect: the metabolic requirements of cell proliferation. *Science*.

[25] Vaupel P., Multhoff G. (2021). Revisiting the Warburg effect: historical dogma versus current understanding. *The Journal of Physiology*.

[26] Liberti M. V., Locasale J. W. (2016). The warburg effect: how does it benefit cancer cells?. *Trends in Biochemical Sciences*.

[27] Palsson-McDermott E. M., O'Neill L. A. (2013). The Warburg effect then and now: from cancer to inflammatory diseases. *Bioessays*.

[28] Siska P. J., Singer K., Evert K., Renner K., Kreutz M. (2020). The immunological Warburg effect: can a metabolic-tumor-stroma score (MeTS) guide cancer immunotherapy?. *Immunological Reviews*.

[29] Icard P., Shulman S., Farhat D., Steyaert J. M., Alifano M., Lincet H. (2018). How the Warburg effect supports aggressiveness and drug resistance of cancer cells?. *Drug Resistance Updates*.

[30] Li J., Cheng D., Zhu M. (2019). OTUB2 stabilizes U2af2 to promote the Warburg effect and tumorigenesis via the AKT/mTOR signaling pathway in non-small cell lung cancer. *Theranostics*.

